# Pan-cancer assessment of mutational landscape in intrinsically disordered hotspots reveals potential driver genes

**DOI:** 10.1093/nar/gkac028

**Published:** 2022-01-21

**Authors:** Haozhe Zou, Tao Pan, Yueying Gao, Renwei Chen, Si Li, Jing Guo, Zhanyu Tian, Gang Xu, Juan Xu, Yanlin Ma, Yongsheng Li

**Affiliations:** Key Laboratory of Tropical Translational Medicine of Ministry of Education, Hainan Provincial Key Laboratory for Human Reproductive Medicine and Genetic Research, International Technology Cooperation Base ‘China–Myanmar Joint Research Center for Prevention and Treatment of Regional Major Disease’ by the Ministry of Science and Technology of China, Hainan Provincial Clinical Research Center for Thalassemia, The First Affiliated Hospital of Hainan Medical University, College of Biomedical Information and Engineering, Hainan Medical University, Haikou 571199, China; College of Bioinformatics Science and Technology, Harbin Medical University, Harbin, Heilongjiang 150081, China; Key Laboratory of Tropical Translational Medicine of Ministry of Education, Hainan Provincial Key Laboratory for Human Reproductive Medicine and Genetic Research, International Technology Cooperation Base ‘China–Myanmar Joint Research Center for Prevention and Treatment of Regional Major Disease’ by the Ministry of Science and Technology of China, Hainan Provincial Clinical Research Center for Thalassemia, The First Affiliated Hospital of Hainan Medical University, College of Biomedical Information and Engineering, Hainan Medical University, Haikou 571199, China; Key Laboratory of Tropical Translational Medicine of Ministry of Education, Hainan Provincial Key Laboratory for Human Reproductive Medicine and Genetic Research, International Technology Cooperation Base ‘China–Myanmar Joint Research Center for Prevention and Treatment of Regional Major Disease’ by the Ministry of Science and Technology of China, Hainan Provincial Clinical Research Center for Thalassemia, The First Affiliated Hospital of Hainan Medical University, College of Biomedical Information and Engineering, Hainan Medical University, Haikou 571199, China; Hainan Women and Children’s Medical Center, Hainan Medical University, Haikou 571199, China; Key Laboratory of Tropical Translational Medicine of Ministry of Education, Hainan Provincial Key Laboratory for Human Reproductive Medicine and Genetic Research, International Technology Cooperation Base ‘China–Myanmar Joint Research Center for Prevention and Treatment of Regional Major Disease’ by the Ministry of Science and Technology of China, Hainan Provincial Clinical Research Center for Thalassemia, The First Affiliated Hospital of Hainan Medical University, College of Biomedical Information and Engineering, Hainan Medical University, Haikou 571199, China; Key Laboratory of Tropical Translational Medicine of Ministry of Education, Hainan Provincial Key Laboratory for Human Reproductive Medicine and Genetic Research, International Technology Cooperation Base ‘China–Myanmar Joint Research Center for Prevention and Treatment of Regional Major Disease’ by the Ministry of Science and Technology of China, Hainan Provincial Clinical Research Center for Thalassemia, The First Affiliated Hospital of Hainan Medical University, College of Biomedical Information and Engineering, Hainan Medical University, Haikou 571199, China; Key Laboratory of Tropical Translational Medicine of Ministry of Education, Hainan Provincial Key Laboratory for Human Reproductive Medicine and Genetic Research, International Technology Cooperation Base ‘China–Myanmar Joint Research Center for Prevention and Treatment of Regional Major Disease’ by the Ministry of Science and Technology of China, Hainan Provincial Clinical Research Center for Thalassemia, The First Affiliated Hospital of Hainan Medical University, College of Biomedical Information and Engineering, Hainan Medical University, Haikou 571199, China; Key Laboratory of Tropical Translational Medicine of Ministry of Education, Hainan Provincial Key Laboratory for Human Reproductive Medicine and Genetic Research, International Technology Cooperation Base ‘China–Myanmar Joint Research Center for Prevention and Treatment of Regional Major Disease’ by the Ministry of Science and Technology of China, Hainan Provincial Clinical Research Center for Thalassemia, The First Affiliated Hospital of Hainan Medical University, College of Biomedical Information and Engineering, Hainan Medical University, Haikou 571199, China; College of Bioinformatics Science and Technology, Harbin Medical University, Harbin, Heilongjiang 150081, China; Key Laboratory of Tropical Translational Medicine of Ministry of Education, Hainan Provincial Key Laboratory for Human Reproductive Medicine and Genetic Research, International Technology Cooperation Base ‘China–Myanmar Joint Research Center for Prevention and Treatment of Regional Major Disease’ by the Ministry of Science and Technology of China, Hainan Provincial Clinical Research Center for Thalassemia, The First Affiliated Hospital of Hainan Medical University, College of Biomedical Information and Engineering, Hainan Medical University, Haikou 571199, China; Key Laboratory of Tropical Translational Medicine of Ministry of Education, Hainan Provincial Key Laboratory for Human Reproductive Medicine and Genetic Research, International Technology Cooperation Base ‘China–Myanmar Joint Research Center for Prevention and Treatment of Regional Major Disease’ by the Ministry of Science and Technology of China, Hainan Provincial Clinical Research Center for Thalassemia, The First Affiliated Hospital of Hainan Medical University, College of Biomedical Information and Engineering, Hainan Medical University, Haikou 571199, China; Hainan Women and Children’s Medical Center, Hainan Medical University, Haikou 571199, China

## Abstract

Large-scale cancer genome sequencing has enabled the catalogs of somatic mutations; however, the mutational impact on intrinsically disordered protein regions (IDRs) has not been systematically investigated to date. Here, we comprehensively characterized the mutational landscapes of IDRs and found that IDRs have higher mutation frequencies across diverse cancers. We thus developed a computational method, ROI-Driver, to identify putative driver genes enriching IDR and domain hotspots in cancer. Numerous well-known cancer-related oncogenes or tumor suppressors that play important roles in cancer signaling regulation, development and immune response were identified at a higher resolution. In particular, the incorporation of IDR structures helps in the identification of novel potential driver genes that play central roles in human protein–protein interaction networks. Interestingly, we found that the putative driver genes with IDR hotspots were significantly enriched with predicted phase separation propensities, suggesting that IDR mutations disrupt phase separation in key cellular pathways. We also identified an appreciable number of clinically relevant genes enriching IDR mutational hotspots that exhibited differential expression patterns and are associated with cancer patient survival. In summary, combinations of mutational effects on IDRs significantly increase the sensitivity of driver detection and are likely to open new therapeutic avenues for various cancers.

## INTRODUCTION

Large-scale cancer genome sequencing studies have generated comprehensive catalogs of mutations for various types of cancer (1,2). However, only a handful of ‘driver’ mutations are considered to provide selective advantages to cancer cells, and the majority of mutations are neutral ‘passengers’ (3). Distinguishing driver mutations from passenger mutations is thus critical to elucidating the underlying mechanism of cancer development and progression.

A majority of cancer-driver detection methods identify significantly mutated genes based on the recurrence of mutations (4,5). However, the presence of rare somatic mutations and limited cohort sizes usually make frequency-based driver identification very challenging (6). In addition, emerging computational algorithms attempt to predict pathogenic mutations based on the effects of mutations on the stability of protein structure (7). Typically, changes in folding free energy are employed in quantifying the magnitude of a mutation’s effect on protein structure stability (8). Most of these methods [i.e. MuStab (9), I-Mutant (10) and PoPMuSiC (11)] incorporate different physicochemical properties and structural preferences of proteins and are trained on differences in folding free energy caused by various mutations. In addition, several methods employ 3D protein structures to identify mutational hotspots in cancer-related genes (12–14). These methods have suggested that including protein structures significantly increases the sensitivity of driver detection in cancer.

Moreover, proteins usually exhibit a continuum of structures and fully folded proteins only represent ∼37% of the human proteome (15). The majority of human proteins contain both folded protein domains and intrinsically disordered regions (IDRs) (16). It has been established that unstructured IDRs in proteins are equally crucial elements for protein function (17,18). However, current research focuses on mutations in folded domain regions with little consideration of mutations in IDRs. Recent studies have demonstrated that IDRs are enriched in disease-associated proteins (19) and ∼25% of disease mutations are located within IDRs (20). These observations raise the important question of how to better predict the pathogenic mutations by incorporating IDRs in cancer.

To address these questions, we hereby propose a computational method to accurately predict potential driver genes or mutations in IDRs across various cancer types. We found that mutations are prevalent in IDRs in cancer, and genes enriched with mutations in IDRs are associated with cancer development. Functional analysis revealed that the potential driver genes play important roles in cancer signaling pathways. In particular, genes enriched with IDR mutations are associated with phase separation, a physical process often mediated by IDRs. Ultimately, considering the impact of DNA mutations on IDRs improves our understanding of complex genetic diseases.

## MATERIALS AND METHODS

### Somatic mutations among various cancers

Genome-wide somatic mutations over 10 000 tumors across 33 different cancer types were obtained from The Cancer Genome Atlas (TCGA) (Supplementary Table S1) (21). The mutational file (MC3) generated by the MC3 working group was used in this study. Seven mutation-calling algorithms with scoring and artifact filtering were utilized to obtain mutations (22). In this study, we only analyzed single-nucleotide polymorphism (SNP) missense mutations. The mutation frequency of genes was calculated as the proportion of samples with mutations in a specific cancer.

### Protein sequences

The sequences of all human proteins were obtained from GENCODE (23) (https://www.gencodegenes.org/). For genes with multiple protein sequences, we selected the longest sequence for further analysis.

### Identification of IDRs and domains in proteins

All protein domains were assigned using Pfam HMM models based on HMMER (http://hmmer.org/). The protein sequences from GENCODE were subjected to this tool for predicting domains in each protein. The remaining significant matches (those with *E*-values <0.0001) were subjected to further analysis (24). In total, 57 599 domains in 15 201 proteins were identified.

To predict the IDRs in a protein, we used IUPred2A that allows energy estimation-based predictions for ordered and disordered residues (25). To avoid confusion between domains and IDRs, we excluded regions that were predicted as both IDRs and domains. Finally, we identified 229 313 IDRs in 12 541 proteins for further analysis.

### ROI-Driver: prioritization of mutated IDR and domain hotspots

We propose a computational method, ROI-Driver, for the prioritization of regions of interest (ROIs) that are enriched with cancer mutations. A protein region with significant enrichment for mutations across individuals is defined as a hotspot. For each ROI in genes, we assume that the observed number of mutations for an ROI follows a binomial distribution (26). The binomial is $( {N,{p_{{\rm ri}}}} )$, in which *N* is the total number of mutations observed in one gene and ${p_{{\rm ri}}}$ is the expected mutation rate for the ROI. The null hypothesis is that the region is not recurrently mutated. We defined ${L_{{\rm ROI}}}$ as the length of the ROI, and ${L_{\rm g}}$ is the length of the gene. For each ROI, we calculated the *P*-value, which is the probability of observing *>=k* mutations in the ROI out of *N* total number of mutations observed in the gene:

$$\begin{eqnarray*}P\left( {X \ge k} \right) &=& 1 - P(X < k)\nonumber\\ && = \ 1 - \mathop \sum \limits_{x\ = \ 0}^{k - 1} \left( {\frac{N}{x}} \right)p_{{\rm ri}}^x{\left( {1 - {p_{{\rm ri}}}} \right)^{N - x}},\end{eqnarray*}$$

where ${p_{{\rm ri}}} = {{{L_{{\rm ROI}}}}}/{{{L_{\rm g}}}}$. In addition, we calculated the enrichment ratio for each ROI as follows:

$$\begin{equation*}{\rm{\ }}{E_{{\rm ROI}}} = \frac{k}{{N\times{L_{{\rm ROI}}}/{L_{\rm g}}}}. \end{equation*}$$

The *P*-values were adjusted and ROIs with *P*_adjusted_ < 0.05, *P* < 0.01 and *E* > 2 were identified as significant ROIs. Only ROIs with >3 mutations were analyzed in this study.

### Enrichment analysis of cancer-related genes

To investigate whether the prioritized genes are enriched in cancer-related genes, we first downloaded known cancer genes from the COSMIC Cancer Gene Census (CGC) (27) and CancerMine (28). Approximately 705 and 4179 genes were obtained from the two databases, respectively. The number of overlapping genes was calculated, and the significance of the overlap was evaluated by random tests. We randomly selected the same number of genes as the prioritized ROI genes 100 000 times. The number of overlapping genes was calculated, and *P*-values were defined as the number of random conditions with a higher number of overlapping genes than observed. Moreover, the observed/expected (O/E) ratio was calculated as follows:

$$\begin{equation*}{\rm{O}}/{\rm{E\ }} = \frac{n}{{\left( {M\times K} \right)/N}}, \end{equation*}$$

where *n* is the number of overlapping genes, *M* and *K* are the number of prioritized genes and cancer-related genes, respectively, and *N* is the total number of protein-coding genes. The codes of ROI-Driver are available at https://github.com/ComputationalEpigeneticsLab/ROI-Driver.

### Phase separation-related proteins

We used PScore to predict the phase separation-related proteins (29). PScore returns a score reflecting the *Z*-score ‘distance’ from values of folded protein sequences, with values ≥4 providing a strong prediction for phase separation (15,30). Proteins with *Z*-score ≥4 were deemed phase separation-related proteins. Next, Fisher’s exact test was used to evaluate whether the prioritized ROI genes were enriched with phase separation-related proteins.

### Tissue-enriched genes

Tissue-enriched (TE) genes were collected from one recent study (31). Briefly, four widely available transcriptome datasets were collected, including the Genotype-Tissue Expression Consortium, Human BodyMap 2.0, Human Protein Atlas and FANTOM5 project. To identify TE genes in each resource, we identified genes that have at least 5-fold higher expression levels in one tissue compared with all other tissues. TE genes in our analysis were refined to those identified in the same tissue from at least two resources.

### Functional analysis of potential cancer drivers

To identify the functions of prioritized genes with ROI mutations, we used clusterProfiler to perform function enrichment analysis (32). Gene Ontology (GO) biological processes were considered in our analysis. We considered GO terms with genes ranging from 15 to 500. The biological processes with *P* < 0.01 and *P*_adjusted_ < 0.05 were considered significant. Next, GO terms were clustered based on the simplifyEnrichment R package (33). Similarities among GO terms were calculated by the ‘GO_similarity’ function, and the cluster results were visualized by the ‘simplifyGO’ function.

### Gene set enrichment analysis

To identify the perturbed pathways disrupted by mutations in IDRs, gene set enrichment analysis (GSEA) was performed (34). First, patients were divided into two groups based on mutations within versus outside IDRs. All protein-coding genes were ranked based on *S* scores, which were calculated as follows:

$$\begin{equation*}S \left( i \right) = \ - \log \left( p \right)\times {\rm sign}\left( {\log\left( {{\rm fold \, change}\left( i \right)} \right)} \right),\end{equation*}$$

where *p* is the Wilcoxon’s rank-sum *P*-value for comparing the expression difference between two groups and fold change is the average expression of the IDR group divided by the average expression within versus outside IDR group. Genes were subjected to pre-ranked GSEA, and cancer hallmark pathways from MSigDB were considered (35).

### Topological features of genes in the human protein–protein interaction network

The topological features of genes in the human protein–protein interaction (PPI) network were calculated based on the igraph package (http://igraph.org/). Here, human PPIs were obtained from HuRI (36). The human PPIs include 52 068 interactions among 8245 proteins. Moreover, we downloaded the PPIs from HumanNet V3, which encompasses 99.8% of human protein-coding genes (37). Three-tier models were used in our analysis, including HumanNet-PI (633 460 interactions among 17 849 genes), HumanNet-FN (977 495 interactions among 18 459 genes) and HumanNet-XC (1 125 494 interactions among 18 462 genes). Three types of topological features, including degree, betweenness and closeness, of each protein were calculated. We next compared the topological features between putative drivers and other proteins by Wilcoxon’s rank-sum test.

### Differential expression of genes

Gene expression profiles of 33 cancer types were downloaded from the TCGA project (38). Only 18 cancer types with >= 5 normal samples were analyzed in this study. Genes that were not expressed in >30% of samples were excluded. We next used Wilcoxon’s rank-sum test to evaluate the expression differences between cancer and normal samples. Genes with fold changes >1 and *P*_adjusted_ ≤ 0.05 were upregulated and fold changes <1 and *P*_adjusted_ ≤ 0.05 were downregulated. Moreover, we also used the same method to evaluate whether cancer patients with ROI mutations versus other patients showed differential gene expression. Genes that were with ≥3 ROI mutations in patients were considered in this analysis.

### Clinical association analysis of genes in cancer

To evaluate the association between gene expression and patient survival, all patients were divided into two groups based on the expression of genes of interest. The log-rank test was used to evaluate the difference in survival rate between the two groups. Moreover, we also divided patients into three groups, including patients with mutations in ROI in a specific gene, patients with gene mutations outside the ROI and patients without mutations in this gene.

## RESULTS

### IDRs are prevalently mutated across cancer genomes

**Figure 1.**
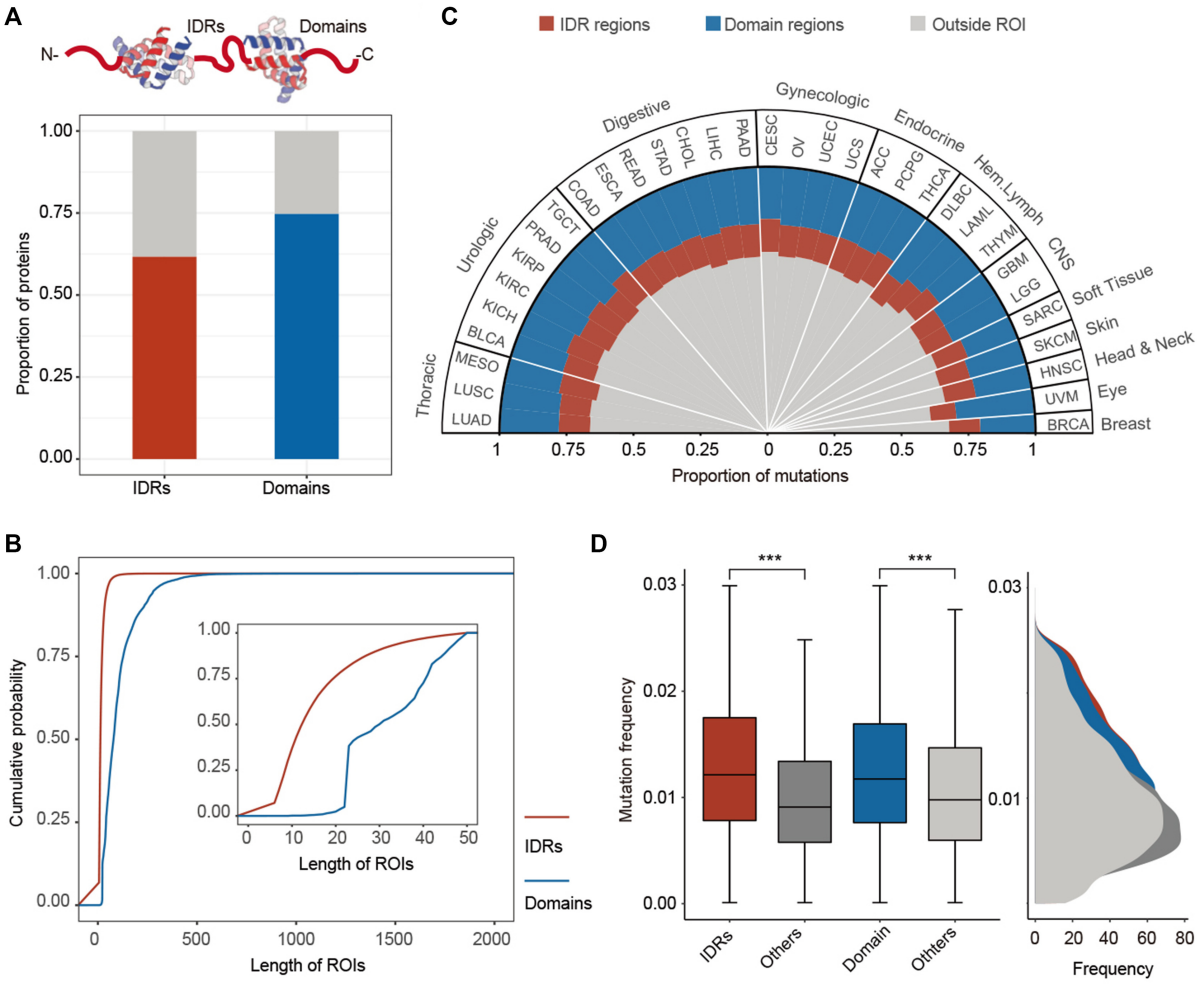
Prevalent mutations in IDRs across cancer types. (**A**) The proportion of proteins with IDR and domain structures. (**B**) The cumulative distribution of the length of IDRs and domains. Inset shows the enlarged region of length <50 amino acids. (**C**) Proportion of mutations located in IDRs or domains across cancer types. (**D**) Frequency of mutations located within versus outside IDRs, and mutations located within versus outside domains. ***P < 0.001, Wilcoxon’s rank-sum test.

Proteins exhibit a continuum of structures ranging from fully folded to entirely intrinsically disordered proteins. We first predicted IDRs and domains in proteins and found that ∼63% of the proteins included at least one IDR structure (Figure 1A). Moreover, the majority (∼75%) of proteins contain at least one domain. Many of these proteins contain both IDRs and domains, including several oncogenes (i.e. ASH1L, CTNNB1 and FNDC1) and tumor suppressors (i.e. TP53, NOTCH2 and EGFR). Next, we calculated the length of the IDRs and domains in each protein. We found that the lengths of domains were significantly longer than IDRs (Figure 1B). The majority of IDRs had lengths ranging from 10 to 50 amino acids.

Lines of evidence have demonstrated that proteins contain IDRs that enrich in disease-associated proteins (19,39). We next mapped all mutations across 33 cancer types to proteins and found that ∼30–40% of mutations occurred within either IDRs or domains (Figure 1C). These observations raise important questions of whether these mutations are correlated to cancer development. Traditionally, candidate driver genes or mutations have been identified by a frequency-based approach, where genes with many recurrent mutations are likely to be associated with cancer (40). We thus calculated the mutation frequency in pan-cancer and found that the mutations located within IDRs or domains had significantly higher frequencies than other mutations (Figure 1D, *P*-values <0.001, Wilcoxon’s rank-sum tests). Moreover, we observed similar results in individual cancer types (Supplementary Figure S1A and B). These results suggest that the mutations can impact IDRs and domains across cancer types.

### Identification of potential drivers with IDR hotspots

**Figure 2.**
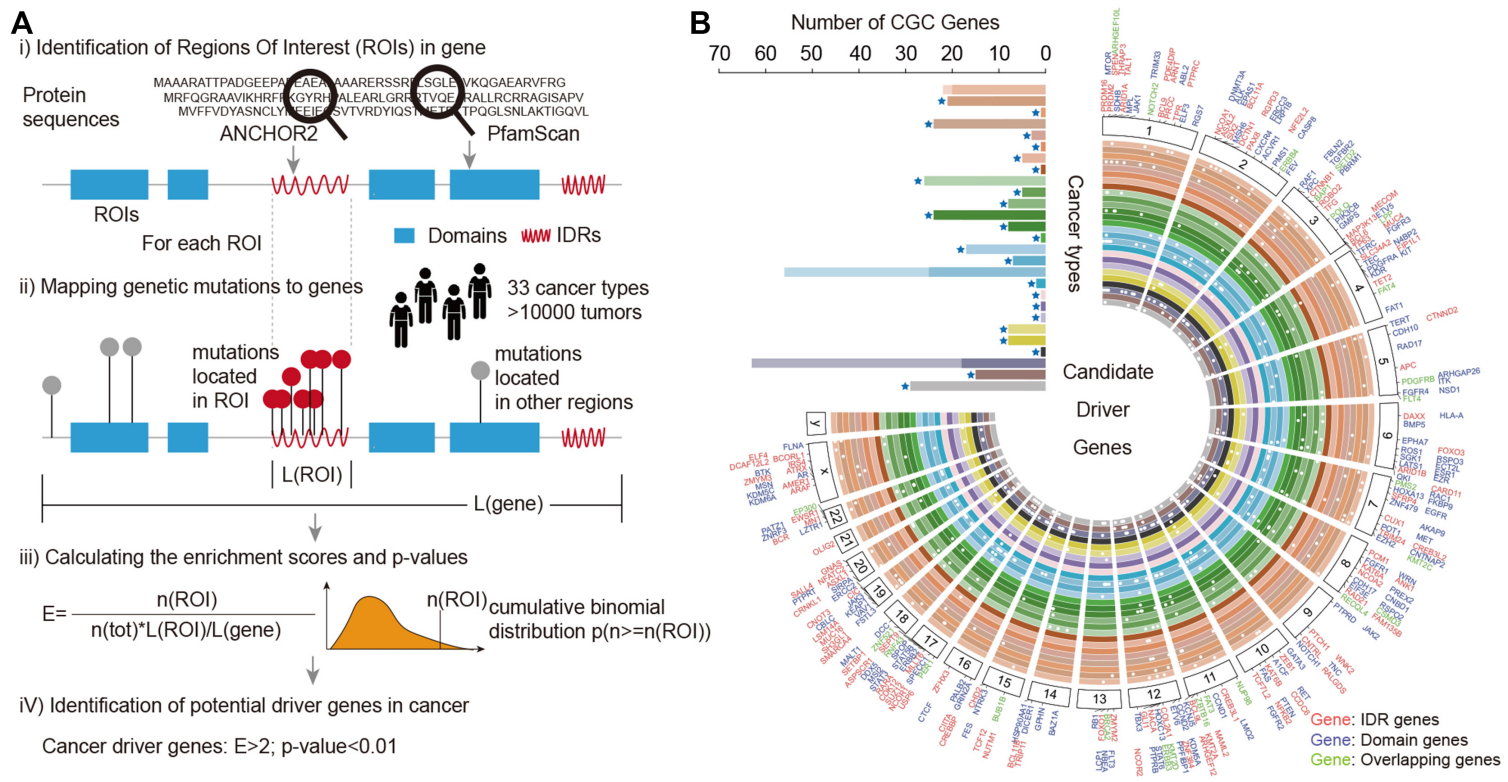
Putative driver gene identification in cancer. (**A**) Workflow of ROI-Driver for identifying the putative driver regions, IDRs or domains. Four main steps were included by integrating mutations with protein structures. (**B**) Circos plot showing the putative driver genes and mutations across cancer types. Bar plot showing the number of driver genes that overlapped with COSMIC genes. Dark colors indicate IDRs and light colors represent domains. Stars indicate that the number of genes enriched with mutations within domains is higher than that in IDRs. The order of cancers from inner to outer is as follows: BRCA, UVM, HNSC, SARC, LGG, GBM, THYM, LAML, DLBC, THCA, PCPG, ACC, UCS, UCEC, OV, CESC, PAAD, LIHC, CHOL, STAD, READ, ESCA, COAD, TGCT, PRAD, KIRP, KIRC, KICH, BLCA, MESO, LUSC and LUAD.

Numerous computational methods have attempted to predict pathogenic mutations based on the characteristics of folded protein regions. However, studies on the impact of mutations with IDRs are limited. We thus developed a computational method, ROI-Driver, to predict the pathogenic mutations in cancer based on the enrichment of mutations in ROIs. This method mainly contains four steps (Figure 2A) that integrate protein structures with genome-wide mutations. Here, we only considered the SNP missense mutations in 33 cancer types. First, IDRs and domains were predicted based on protein sequences. Enrichment and significance were evaluated by considering the number of mutations in the ROIs and the relative length of ROIs to the entire gene. IDRs or domains in genes with *P*_adjusted_ < 0.05, *P* < 0.01 and *E* > 2 were identified as significant ROIs, i.e. hotspots in cancer.

We next applied our workflow to identify significantly mutated IDR or domain hotspots for each cancer cohort. In total, we identified 1–919 IDRs and 1–423 domains enriched by missense mutations (Supplementary Figure S2A and B, and Supplementary Tables S2 and S3). These IDRs and domains involved 1–818 and 1–375 genes in 33 cancer types, respectively (Supplementary Figure S2C and D). Next, we particularly focused on cancer-related genes from CGC (27) and CancerMine (28). We found that 1–63 genes in CGC were prioritized by mutation impact on IDRs across cancer types (Figure 2B). Moreover, several genes were enriched by mutations in both IDRs and domains, such as ERBB4, NOTCH2, FLT4, EP300 and BRCA2. Similarly, we obtained 519 genes that were enriched with mutations in IDRs and 662 genes enriched with mutations in domains, as well as 96 genes enriched with mutations in both IDRs and domains in CancerMine (Supplementary Figure S3). Together, we have identified prevalent cancer-related mutations in IDRs and utilize these in identifying the potential driver genes.

### Known cancer genes harbor IDR hotspots

**Figure 3.**
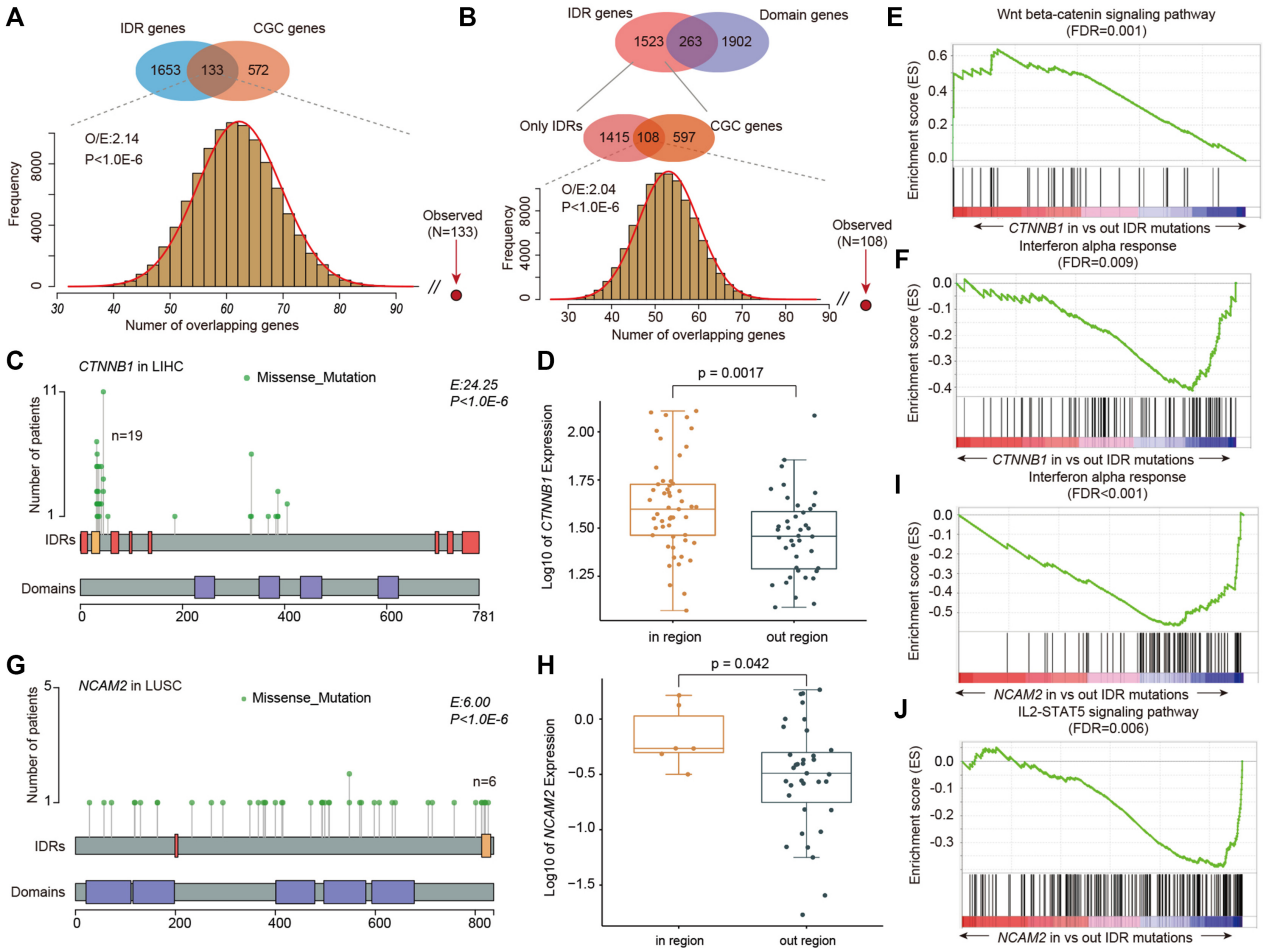
Putative driver genes overlapping with known cancer-related genes. (**A**) Venn plot showing the overlap between IDR hotspots and COSMIC genes. The bar plot at the bottom shows the frequency of the number of overlapping genes in random conditions. (**B**) Venn diagram showing the overlap of IDR hotspots and domain hotspots. The bottom Venn diagram shows the overlap between only IDR hotspots and COSMIC genes. The bar plot shows the distribution in random conditions. (**C**) Lollipop plot showing mutations in the CTNNB1 gene. IDRs and domains are shown at the bottom. (**D**) Boxplot showing the expression of CTNNB1 in patients with mutations within versus outside IDRs. (**E**, **F**) GSEA plots showing the activation of the Wnt signaling pathway and repression of interferon-alpha response. (**G**) Lollipop plot showing the mutations in the NCAM2 gene. IDRs and domains are shown at the bottom. (**H**) Boxplot showing the expression of NCAM2 in patients with mutations within versus outside IDRs. (**I**, **J**) GSEA plots showing the repression of interferon-alpha response and IL2–STAT5 signaling pathway in patients with NCAM2 IDR mutations.

We next compared our set of predicted driver genes to the set of curated genes in COSMIC and CancerMine. Overall, our workflow identified many additional genes (1653 genes) with IDR hotspots compared with COSMIC (Figure 3A). In total, 133 genes prioritized in our method were verified in COSMIC, including several well-known oncogenes and tumor suppressors (Table 1). We next randomly selected the same number of genes as our workflow prioritized. We found that the genes identified in our workflow significantly overlapped with COSMIC (Figure 3A, O/E = 2.14 and *P*-value <1.0E−6). We obtained similar results in the CancerMine dataset (Supplementary Figure S4). As previous studies involved predicting drivers based on mutation-enriched domains, we compared genes enriching IDR hotspots with those enriching domain hotspots. We found that 263 genes were prioritized by both IDR and domain hotspots, and 1523 genes were only identified by IDR mutation enrichment (Figure 3B). Among these genes, 108 genes overlapped with driver genes in COSMIC, which was significantly higher than random conditions (Figure 3B, O/E = 2.04 and *P*-value <1.0E−6).

**Table 1.** Top 10 putative driver genes and mutations with IDR mutational hotspots across cancer types

Genes	Cancers	*E*-value	*P*-value	Resource	Potential driver mutations
ANK1	SARC	32.52	1.02E−6	CGC	p.L1626M|p.L1626Q|p.A1621D
CTNNB1	PRAD	27.56	2.62E−10	CGC/CancerMine	p.S33C|p.D32Y|p.S33Y|p.S37A|p.D32V|p.D32H
GLI1	STAD	25.14	4.30E−6	CGC/CancerMine	p.R81W|p.R81Q|p.S84P
ZMYM2	LUSC	25.04	4.37E−6	CGC/CancerMine	p.G268V|p.M264I|p.Q272H
NOTCH2	LUAD	23.17	7.84E−6	CGC/CancerMine	p.M2183V|p.G2174R|p.L2184F
MUC16	HNSC	22.07	1.22E−5	CGC/CancerMine	p.T3859K|p.R3852G|p.Q3851L
PRDM2	COAD	21.48	1.05E−5	CGC/CancerMine	p.Y1680H|p.S1679I|p.R1683H
TRIP11	SKCM	20.61	1.29E−6	CGC	p.G149W|p.H158Y|p.S145L|p.F146L
PRCC	BLCA	18.41	2.75E−6	CGC	p.I245V|p.S243C|p.I245M
DAXX	GBM	17.76	6.34E−6	CGC/CancerMine	p.C664F|p.P667L|p.K658N

In addition, we analyzed the expression data of several prioritized genes to obtain further evidence corroborating the biological validity of candidate driver genes. For example, we prioritized an IDR of CTNNB1 in the liver hepatocellular carcinoma (LIHC). There were 19 missense mutations located within IDRs, which is ∼24.25-fold to that of the whole gene (Figure 3C, *P* < 1.0E−6). We found that patients with IDR mutations showed significantly higher expression than those with mutations outside IDRs (Figure 3D, *P* = 0.0017). Lines of evidence have demonstrated that mutations in CTNNB1 can activate the Wnt signaling pathway and appear to be major events in hepatocellular carcinoma (41–43). Indeed, we performed GSEA based on differential expression patterns between the two groups. We found that genes showing higher expression in patients with IDR mutations were significantly enriched in cancer-related hallmark pathways. In particular, the canonical Wnt signaling pathway was activated (Figure 3E, FDR = 0.001), while the interferon-alpha response pathway was repressed (Figure 3F, FDR = 0.009) in patients with CTNNB1 IDR mutations.

Another example is the NCAM2 gene that is enriched with IDR mutations in lung squamous cell carcinoma (LUSC). We identified six mutations that mapped to an IDR in LUSC (Figure 3G), which showed 6-fold enrichment relative to the whole gene (*P* < 1.0E−6). Expression analysis revealed that patients with mutations in IDRs exhibited significantly higher expression of NCAM2 (Figure 3H, *P* = 0.042). NCAM2 has been identified as a target molecule in several types of cancers (44,45). Functional analysis indicated that mutation in the NCAM2 IDR is associated with repressed immune-related pathways, such as interferon-alpha response (Figure 3I, FDR < 0.001) and IL2–STAT5 signaling pathway (Figure 3J, FDR = 0.006). Moreover, we also identified several genes with IDR hotspots, which play important roles in cancer, such as FNDC1 in lung adenocarcinoma (Supplementary Figure S5A and B) and TANC2 in uterine corpus endometrial carcinoma (Supplementary Figure S5C and D). The upregulated expression of FNDC1 has been demonstrated to be correlated to poor prognosis in cancer (46) and TANC2 was identified as a driver gene in cancer with effects on cell growth, survival and transformation (47). Together, these results suggest that IDR mutations may help identify putative driver genes in cancer.

### IDR hotspots are located in central positions of human PPIs

**Figure 4.**
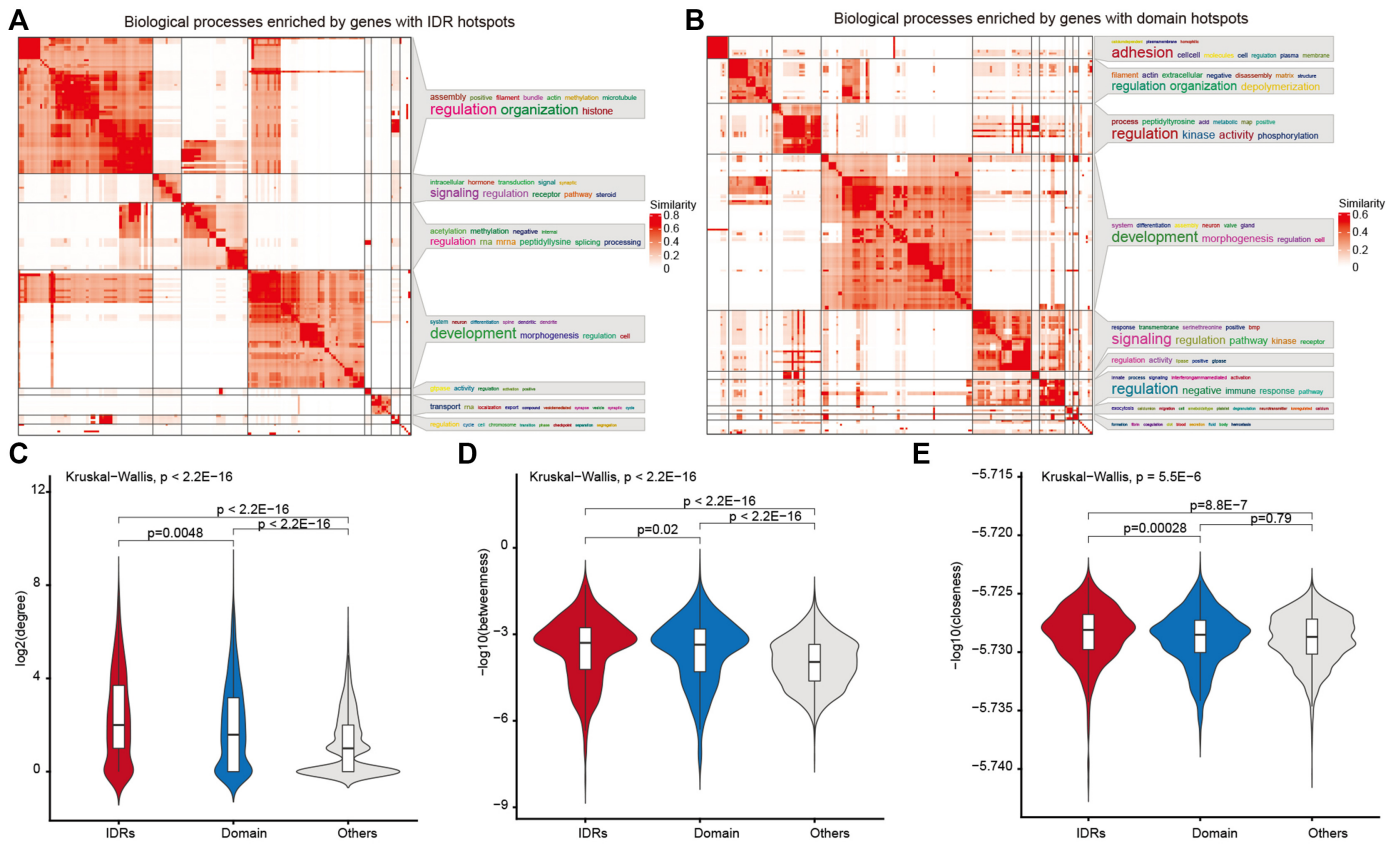
Hotspot genes enriched in cancer-related pathways and central region of the PPI network. Heat maps showing the biological processes enriched by genes with IDR (**A**) or domain (**B**) hotspots. (**C**) Degree distribution of genes with IDR or domain hotspots and other genes. (**D**) Betweenness distribution of genes with IDR or domain hotspots and other genes. (**E**) Closeness distributions of genes with IDR or domain hotspots and other genes.

We next performed GO enrichment analysis for functional annotations of genes with predicted IDR and domain hotspots. Functional enrichment analysis implicates the putative IDR driver genes in diverse biological functions such as signaling regulation, development and cell morphogenesis (Figure 4A). Moreover, the putative domain driver genes were significantly enriched in signaling regulation, development, kinase activity and immune response (Figure 4B).

Cancer genes often function as network hub proteins that are involved in many cellular processes (48–50). We next investigated the topological features of the prioritized driver genes with IDR or domain mutations. As expected, we found that genes with domain hotspots showed significantly higher degrees and betweenness than other genes (Figure 4C–E). Interestingly, we found that genes with IDR hotspots exhibited a significantly higher degree, betweenness and closeness than those genes with domain hotspots (Figure 4C–E). Moreover, we used three other human PPI networks in this analysis. We found that the results were robust to different networks (Supplementary Figure S6). These results indicated that genes with IDR hotspots are located in the central region of human PPI networks and play important roles in diverse biological processes.

### IDR hotspots are associated with phase separation

**Figure 5.**
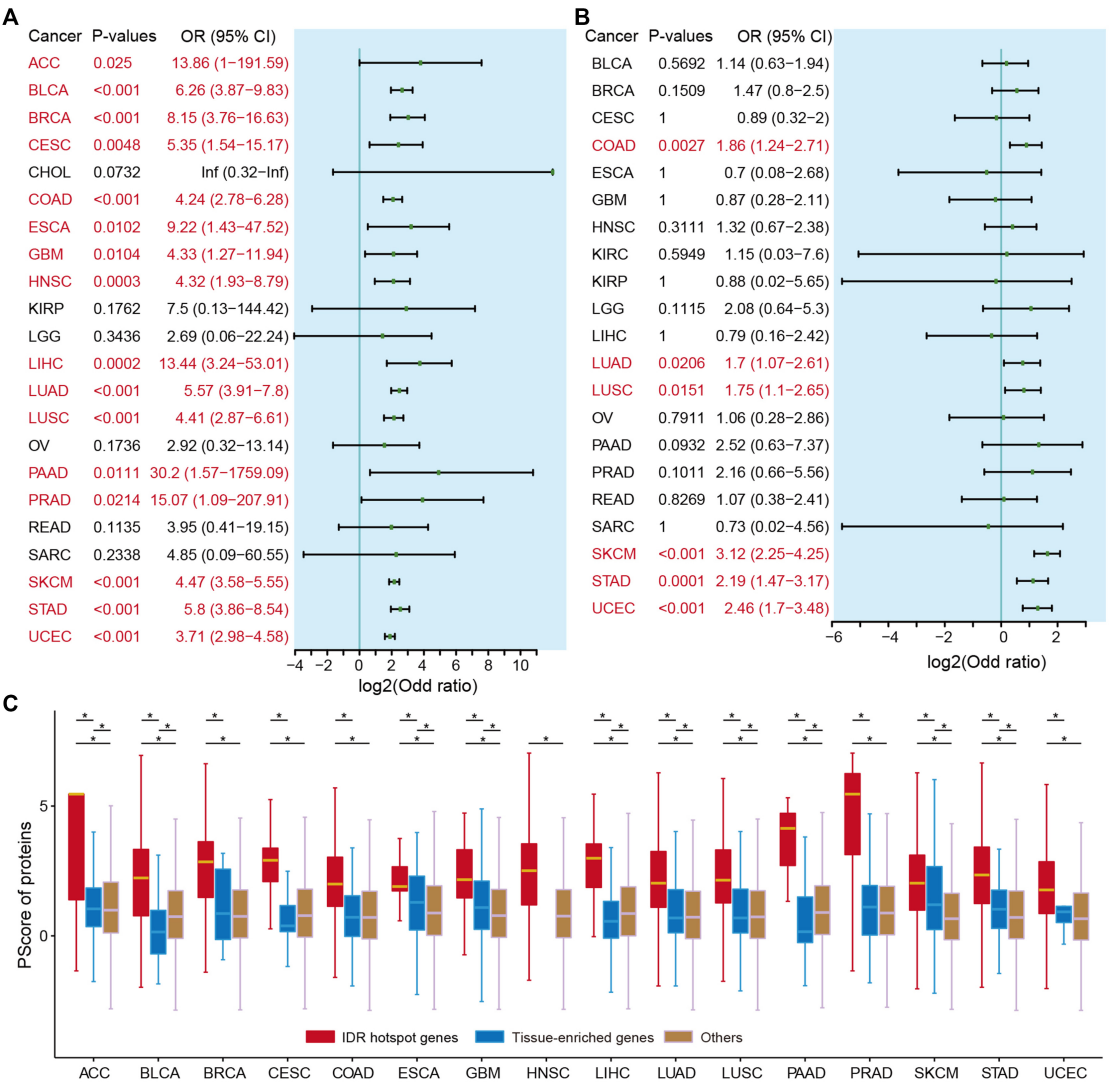
Putative driver genes associated with phase separation. (**A**) Odd ratios of Fisher’s exact tests showing the enrichment of IDR driver genes in phase separation-related genes. (**B**) Odd ratios of Fisher’s exact tests showing the enrichment of domain driver genes in phase separation-related genes. Cancers with *P*-values <0.05 are marked in red. (**C**) Boxplots showing the PScore of proteins encoded by IDR hotspot genes, TE genes and other genes across cancer types. Red, IDR hotspot genes; blue, TE genes; brown, other genes. *P-values <0.01 for Wilcoxon’s rank-sum tests.

As noted earlier, IDRs are important for regulating phase separation (51,52). A previous study has demonstrated significant enrichment for phase separation of proteins associated with autism spectrum disorder and neurological disorders (15). However, the extent of enrichment for IDR or domain mutation in relevant genes related to phase separation is unclear. We thus predicted the phase separation-related proteins based on the PScore method (29). We calculated the proportion of genes that are related to phase separation in each cancer type. Based on Fisher’s exact tests, we found that genes enriched with IDR mutations significantly overlapped with phase separation-related genes in 72.72% (16/22) of cancer types (Figure 5A, *P*-values <0.05). However, only 28.57% (6/21) of the cancer types with genes that showed enrichment of domain mutations significantly overlapped with phase separation-related genes (Figure 5B, *P*-values <0.05).

Moreover, to investigate whether the phase separation enrichment is a property of TE genes, we next obtained the TE genes from a recent study (31). Although TE genes exhibited significantly higher PScores in several tissues than others, the IDR hotspot genes showed the highest PScores across cancer types (Figure 5C). We did not observe similar results in domain hotspot genes (Supplementary Figure S7). These results strongly suggest that phase separation is not a baseline property of TE genes. In contrast, the IDR hotspot genes are associated with phase separation, which may specifically be involved in biological processes underlying cancer development and progression.

### IDR hotspots are associated with differential expression and patient survival

**Figure 6.**
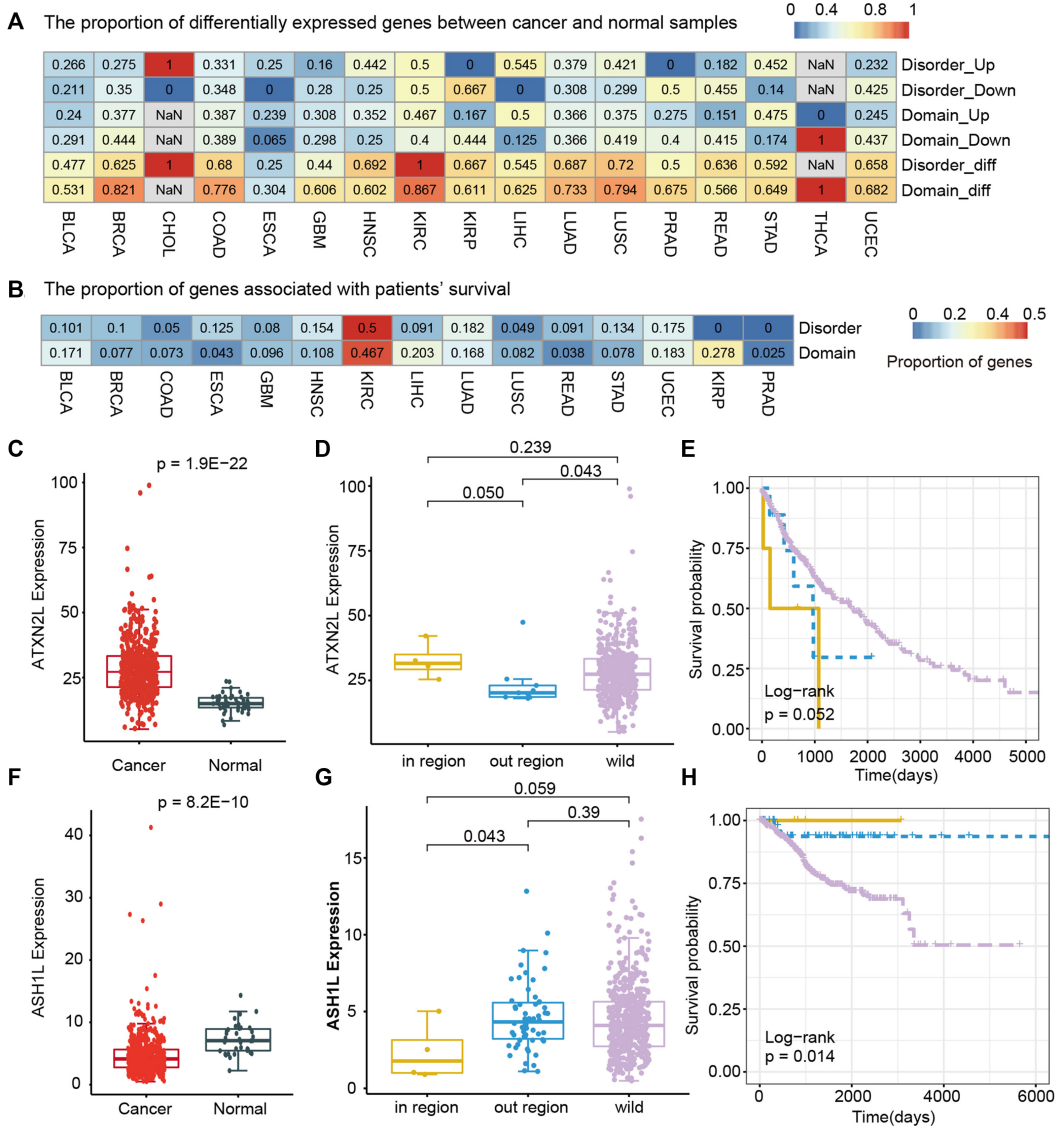
Driver genes associated with expression perturbation and clinical survival. (**A**) Heat map showing the proportion of driver genes with a perturbed expression between cancer and normal samples. (**B**) Heat map showing the proportion of driver genes that are associated with patient survival. (**C**) Boxplot showing the expression of ATXN2L in LUSC and normal samples. (**D**) Boxplot showing the expression in patients with mutations within versus outside IDRs and wild type. (**E**) Survival curves are plotted for LUSC patients with ATXN2L mutations within and outside IDRs and wild type. (**F**) Boxplot showing the expression of ASH1L in UCEC cancer and normal samples. (**G**) Boxplot showing the expression in UCEC patients with mutations within versus outside IDRs and wild type. (**H**) Survival curves are plotted for patients with ASH1L mutations within and outside IDRs and wild type.

Clinical relevance is commonly used to define cancer-related clinical features, including differential expression and association with survival. To further investigate the clinical utility of putative driver genes with IDR or domain hotspots, we identified several clinically relevant drivers (Figure 6A and B). In particular, ∼25% (in ESCA) to 100% (in KIRC and CHOL) of putative driver genes with IDR hotspots exhibited differential expression in various cancers (Figure 6A). In addition, we found that 50% of putative drivers with IDR hotspots and 46.7% of drivers with domain hotspots are correlated to patient survival in KIRC (Figure 6B). For example, ATXN2L was prioritized as a driver gene in LUSC (Supplementary Figure S8A). We found that ATXN2L was significantly upregulated in cancer (Figure 6C, *P* = 1.9E−22), suggesting its oncogenic role. In particular, cancer patients with IDR mutations exhibited even higher expression of ATXN2L than patients with mutations outside IDRs or wild types (Figure 6D). A previous study has demonstrated that ATXN2L upregulated by epidermal growth factor promotes cancer cell invasiveness and oxaliplatin resistance (53). These observations indicate that ATXN2L functions as an oncogene in cancer, and mutations in IDRs further promote carcinogenesis. Clinical survival analysis revealed that patients with IDR hotspots have worse prognosis than other patients (Figure 6E, log-rank test, *P* = 0.052).

Another example is the ASH1L gene (encoding a histone methyltransferase protein), which was prioritized to have IDR hotspots in UCEC (Supplementary Figure S8B). ASH1L has been found to be frequently altered in various cancers (54,55). We found that ASH1L exhibited significantly lower expression in cancer (Figure 6F, *P* = 8.2E−10). Moreover, patients with IDR mutations exhibited significantly downregulated expression than other patients (Figure 6G). These results suggest that the mutations within IDRs might play protective roles in cancer. We next compared the survival rates of three groups of patients and found that patients with mutations in IDR show better survival than others (Figure 6H, log-rank *P* = 0.014). Taken together, these results suggest the clinical relevance of putative driver genes with IDR hotspots.

## DISCUSSION

Although tremendous efforts on genome-wide cancer genome analyses have facilitated the establishment of somatic mutation catalogs in cancer, the identification of driver genes remains a challenge. The impact of IDRs, particularly those that lack a stable folded protein structure, on cancer remains unclear. In this study, we systematically investigated the mutation distribution in IDRs across 33 cancer types and further proposed a computational method to prioritize genes enriched with mutations in IDRs. We observed a higher frequency of mutations in IDRs in cancer-related genes. We also compared our putative driver gene list with well-known cancer-related genes and found that the putative drivers identified by ROI-Driver significantly overlapped with cancer genes. Accordingly, assessing IDR structures will help identify additional cancer genes that may play important roles in cancer-related pathways. We observed a significant enrichment of putative driver genes in signaling regulation, development and immune response, which have previously been implicated in tumor growth. Thus, functional enrichment of putative IDR hotspot driver genes in critical signaling pathways provides clear biological evidence for their roles in cancer.

Moreover, we analyzed mutations in various cancers and prioritized the IDRs and domains in pan-cancer. Furthermore, we identified 395 genes that are enriched with mutations in IDRs and 158 genes enriched with mutations in domains (Supplementary Figure S9). A total of 40 and 131 genes enriched with mutations in IDRs that are annotated in the CGC and CancerMine were found. The prioritized genes were significantly overlapped with known cancer-related genes in the CGC and CancerMine (all *P*-values <0.01). In addition, 384 additional genes were prioritized by IDR mutation enrichment analysis (Supplementary Figure S9). Subsequently, 38 genes annotated as cancer genes in CGC and 126 genes annotated in CancerMine were found to be significantly higher than random conditions (Supplementary Figure S9). We discovered that CTNNB1 also harbored IDR mutation hotspots that merged with pan-cancer (Supplementary Figure S10). These results suggested that incorporating IDR information may help in prioritizing cancer-related genes.

Furthermore, phase separation, which was mediated by IDRs, has lately been recognized for its roles in cellular organization and regulation (56–58). We also observed significantly higher enrichment of our driver genes with genes associated with phase separation, suggesting that IDR mutations disrupt phase separation in key cellular processes. Moreover, PScore only predicted proteins expected to phase separate due to planar pi–pi interactions in their IDRs. Thus, our predictions based on this method were considered conservative estimates. Moreover, because the PScores of putative IDR driver proteins were significantly greater than the PScores of other proteins encoded by highly abundant TE genes (Figure 5C), this observation strongly suggested that phase separation was not a baseline property of highly TE proteins. Rather, phase separation may specifically be involved in the pathways underlying cancer development. We prioritized IDRs enriched with mutations and found that these genes were associated with phase separation; however, not all IDRs contributed to phase separation. Thus, further studies should determine whether an IDR of interest linked to cancer would phase separate by experimental methods.

In the context of identifying the putative driver genes in cancer, protein structure-based detection methods offer significant advantages over approaches limited to protein sequences (59). However, protein structure-based methods suffer from the limited coverage of the human proteome, and numerous proteins have unknown structures (60). Importantly, a growing number of examples of verified IDRs have been collated into several databases such as DisProt (61); these entries only provide a small sample of IDRs. Accordingly, most efforts focused on predicting IDRs in proteins, such as flDPnn (62), DisoRDPbind (63) and ANCHOR2 (25). IUPred2A is one of the most widely used and reliable intrinsic disorder prediction algorithms. We thus used the predicted IDRs from IUPred2A in our analysis. Moreover, we predicted the IDRs based on flDPnn and ESpritz (64) and found that ∼80.35% and ∼65.85% of the IDRs predicted by IUPred2A were supported by ESpritz and flDPnn (Supplementary Figure S11A and B). In particular, ∼94.98% and ∼43.1% of the prioritized IDRs in IUPred2A were supported by ESpritz and flDPnn (Supplementary Figure S11C and D). Significant experimental technical improvements in the cryogenic electron microscopy technique (65) and computational methods are expected to expand the list of structurally resolved proteomes.

Recurrently mutated coding and noncoding regions—such as long intergenic noncoding RNA genes and regulatory and enhancer regions (54)—play important roles in cancer development and progression. Li *et al.* provide a blueprint for the identification and functional validation of cancer-associated mutations in noncoding regions of the genome (66). Moreover, intronic mutations have also been correlated with cancer development (67). Our proposed ROI-Driver pipeline can be easily extended to genome-wide analyses to reveal the landscape of functional mutations within the noncoding genome. Moreover, we identified number of genes that were correlated with patient survival, suggesting their clinical relevance. We next identified the potential candidate drugs whose activities are correlated with the expression of prioritized genes based on Genomics of Drug Sensitivity in Cancer (68). We identified numerous genes that were correlated with drug activities across cancer cell lines (Supplementary Figure S12), providing potential drug targets for further functional validation.

In summary, with the development of new computational tools coupled with established experimental methods, the mutational impact of IDRs can be evaluated to link mutations to functional effects in complex diseases. Additionally, the knowledge of protein IDR mutations can potentially help uncover druggable hotspots in cancer. Such studies will open new therapeutic avenues for various cancers and will provide novel insights into precision medicine in cancer.

## DATA AVAILABILITY

All data and tools used in this study are provided in the ‘Materials and Methods’ section.

## Supplementary Material

gkac028_Supplemental_Files
