# Supplementary material for: Pan-cancer assessment of mutational landscape in intrinsically disordered hotspots reveals potential driver genes
Source: Nucleic Acids Res. 2022 Jan 21;50(9):e49. doi: 10.1093/nar/gkac028 (PMC9122534; doi:10.1093/nar/gkac028)
Supplement: gkac028_Supplemental_Files [file gkac028_supplemental_files.zip › Supplementary figures-revision2.pdf]

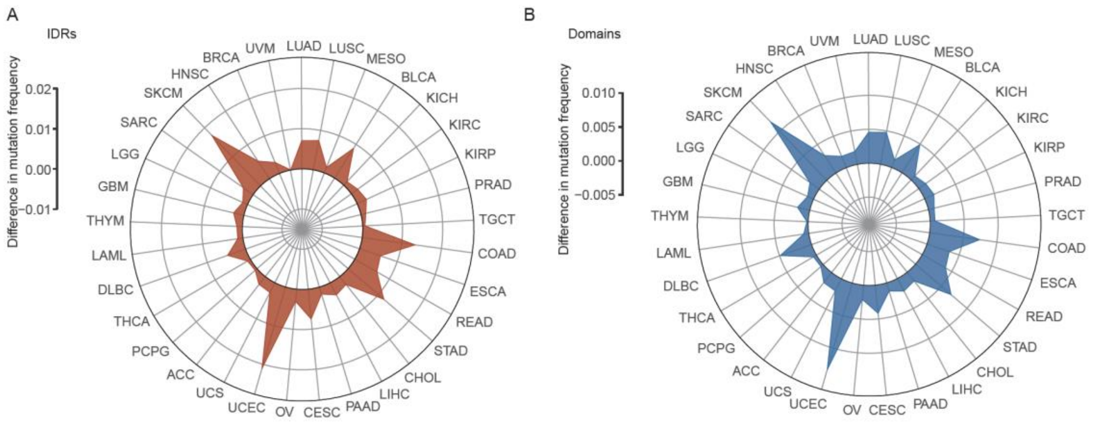

Figure S1. Difference in average frequency for mutations located in IDRs or domains vs. other regions across cancer types. A for IDRs and B for domains.

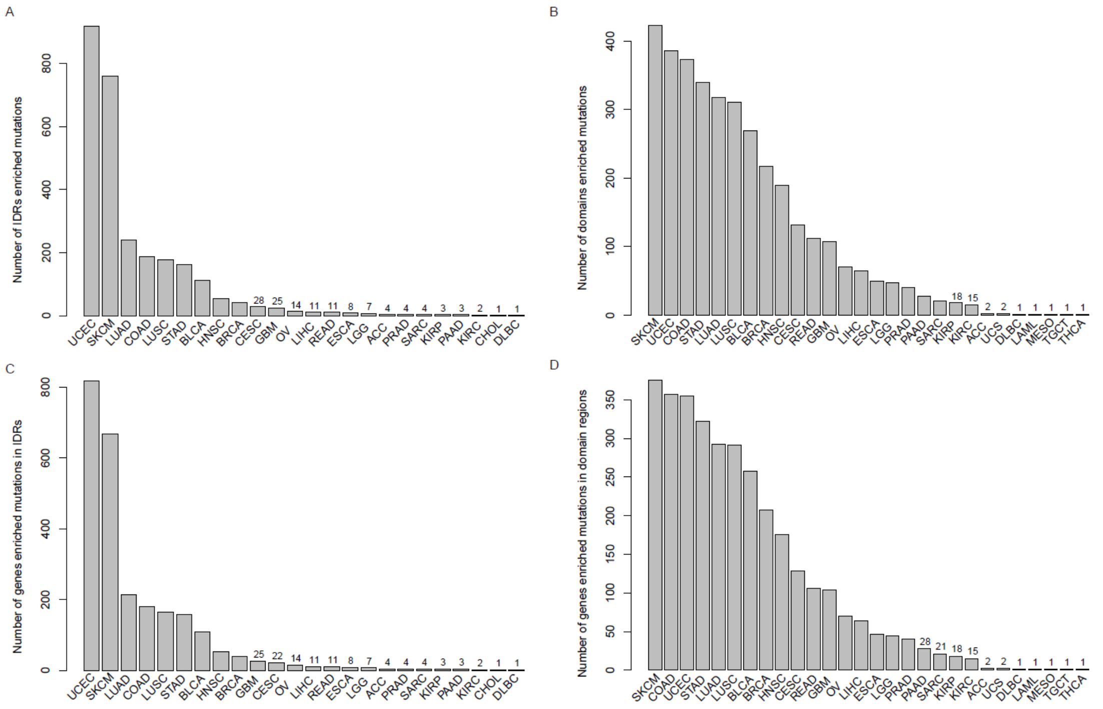

Figure S2. Number of genes or ROIs prioritized in each cancer type. A, Number of IDRs enriched mutations across cancers. B, Number of domains enriched mutations across cancers. C, Number of genes enriched mutations in IDRs. D, Number of genes enriched mutations in domains.

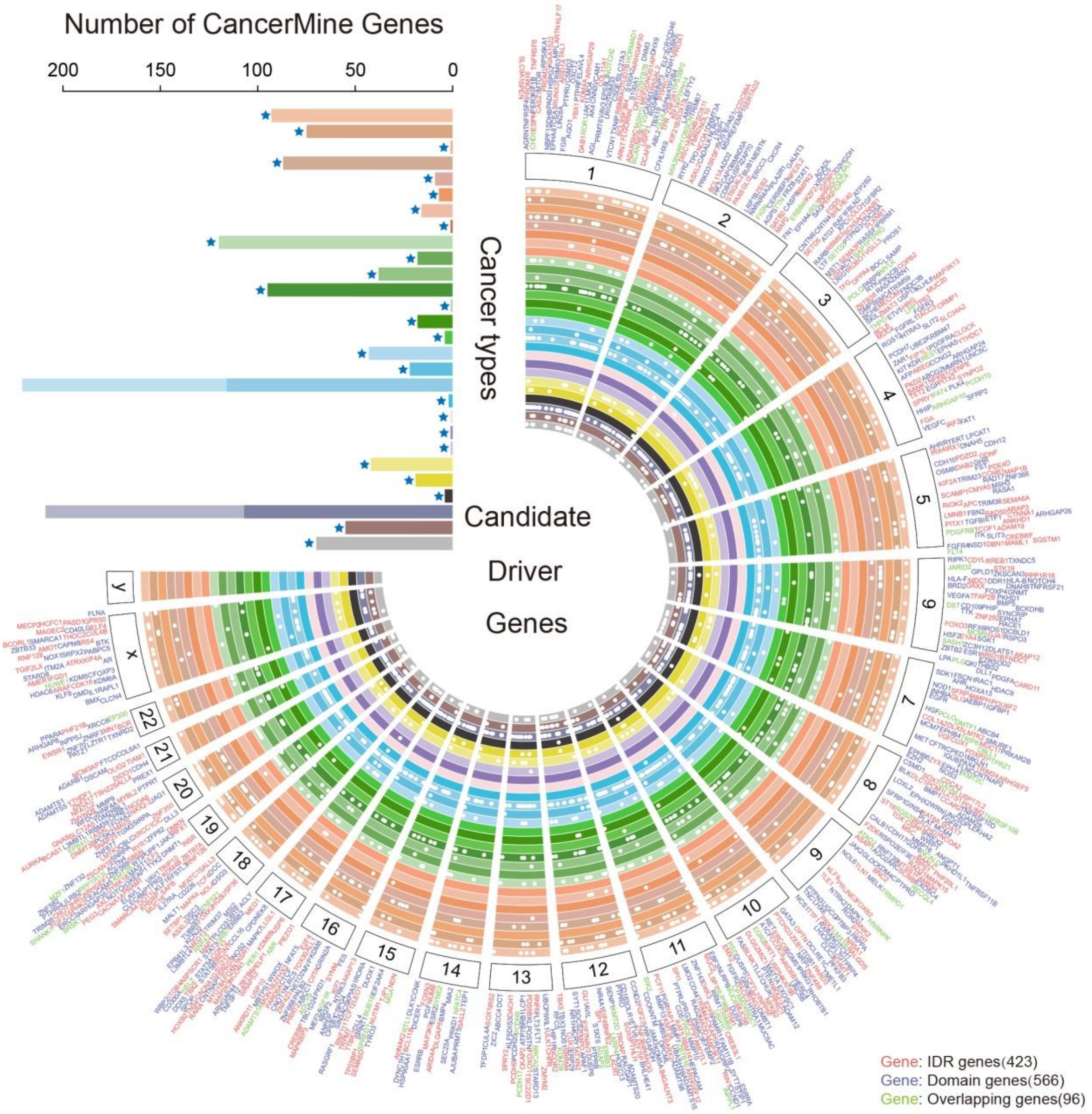

Figure S3. Circos plot showing the putative driver genes and mutations across cancer types. Bar plot showing the number of driver genes and that overlapped with COSMIC genes. The order of cancers from inner to outer is: BRCA, UVM, HNSC, SARC, LGG, GBM, THYM, LAML, DLBC, THCA, PCPG, ACC, UCS, UCEC, OV, CESC, PAAD, LIHC, CHOL, STAD, READ, ESCA, COAD, TGCT, PRAD, KIRP, KIRC, KICH, BLCA, MESO, LUSC and LUAD.

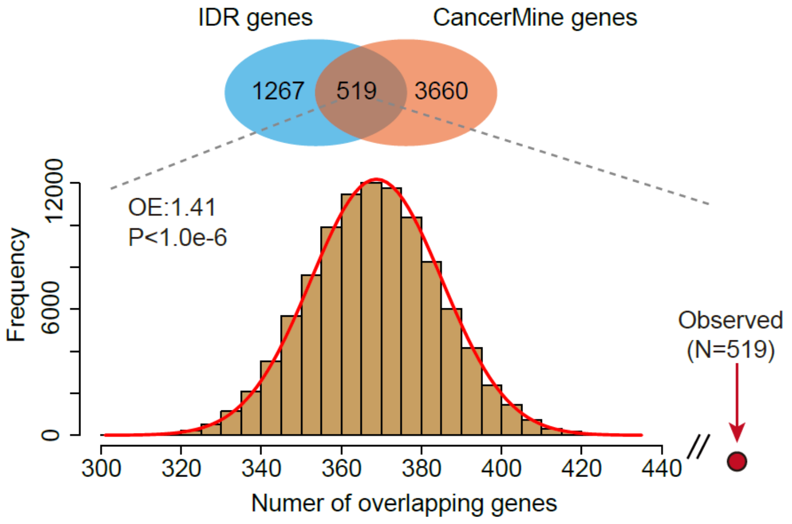

Figure S4. Putative driver genes overlapped with known cancer-related genes. Venn plot showing the overlap between IDR hotspots and CancerMine genes. The bar plot at the bottom showing the frequency of number of overlapping genes in random conditions.

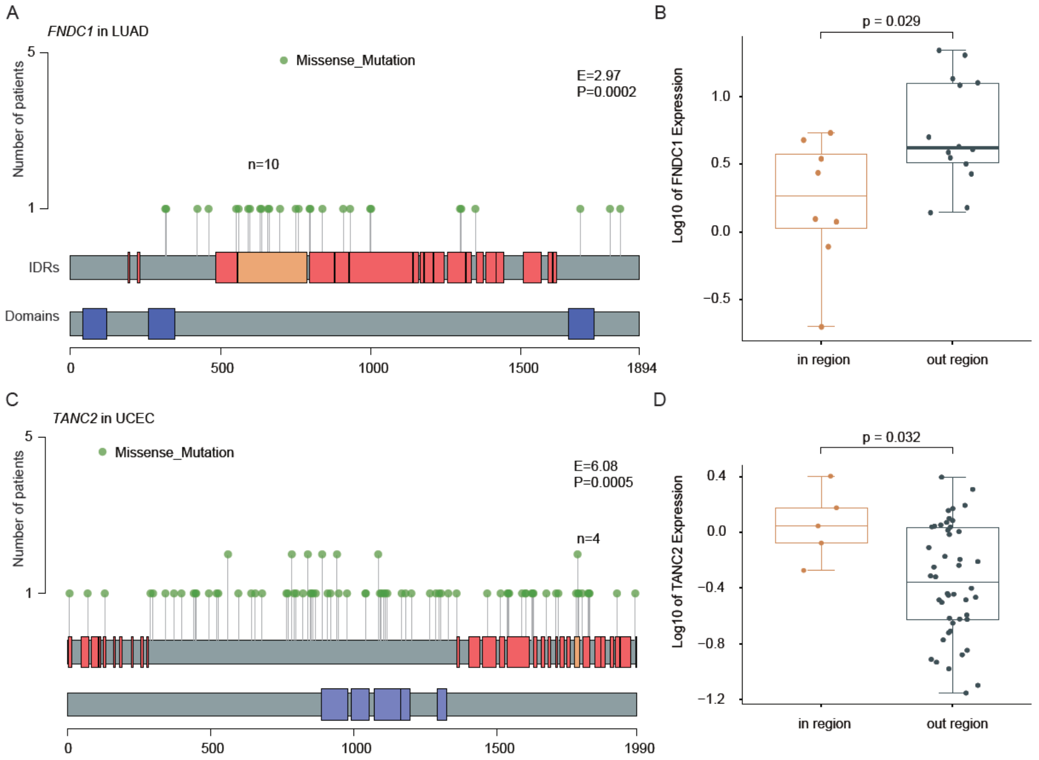

Figure S5. Prioritized genes mutations and expression in cancer. A, Lollipop plot showing the mutations in FNDC1 gene. IDRs and domains were shown in bottom. B, Boxplot showing the expression of FNDC1 in patients with mutations in vs. out IDR. C, Lollipop plot showing the mutations in TANC2 gene. IDRs and domains were shown in bottom. D, Boxplot showing the expression of TANC2 in patients with mutations in vs. out IDR.

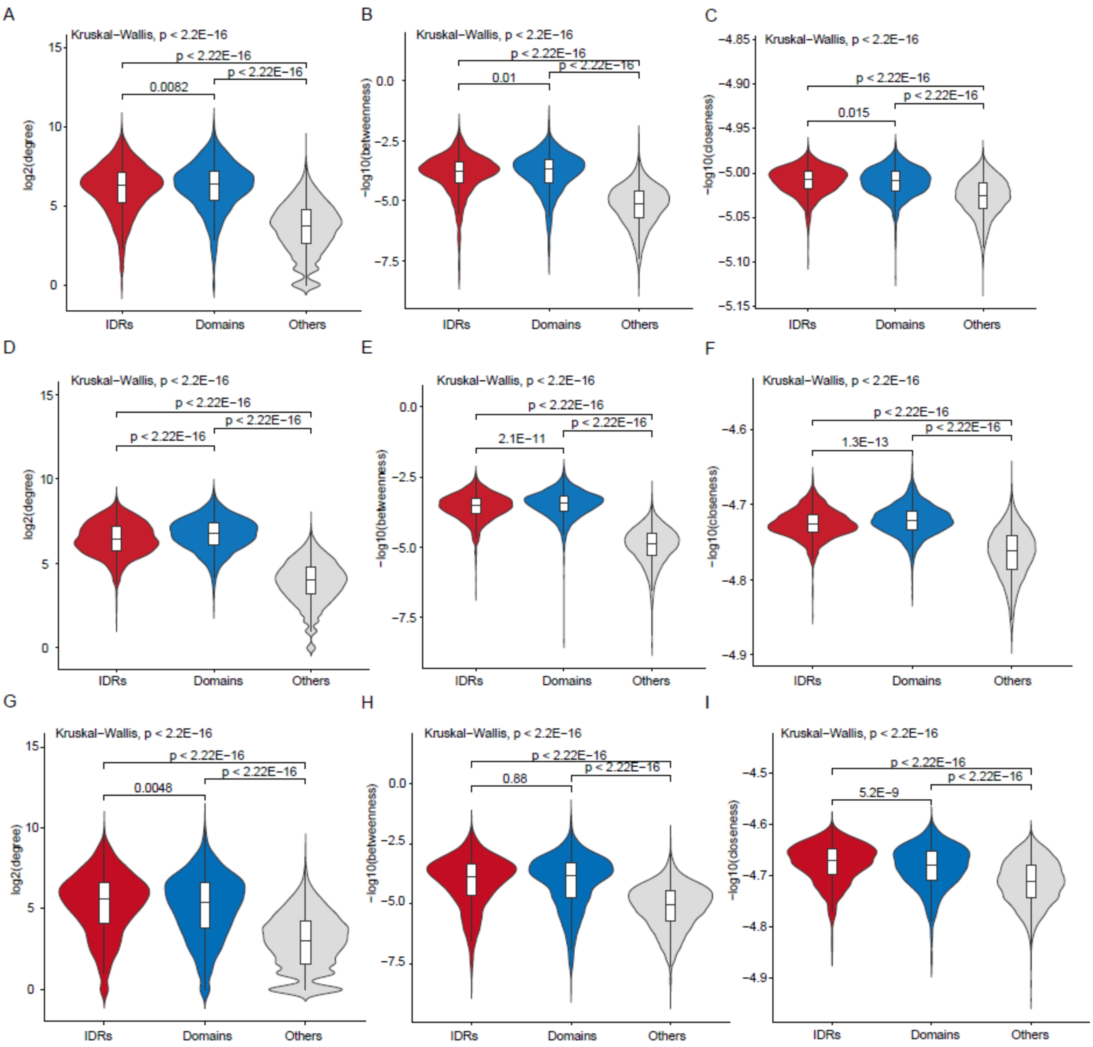

Figure S6. Topological features of genes enriched mutations within IDRs, domains and other genes in PPI networks. A-C for HumanNet-FN; D-F for Human Net-XC and G-I for HumanNet-PI.

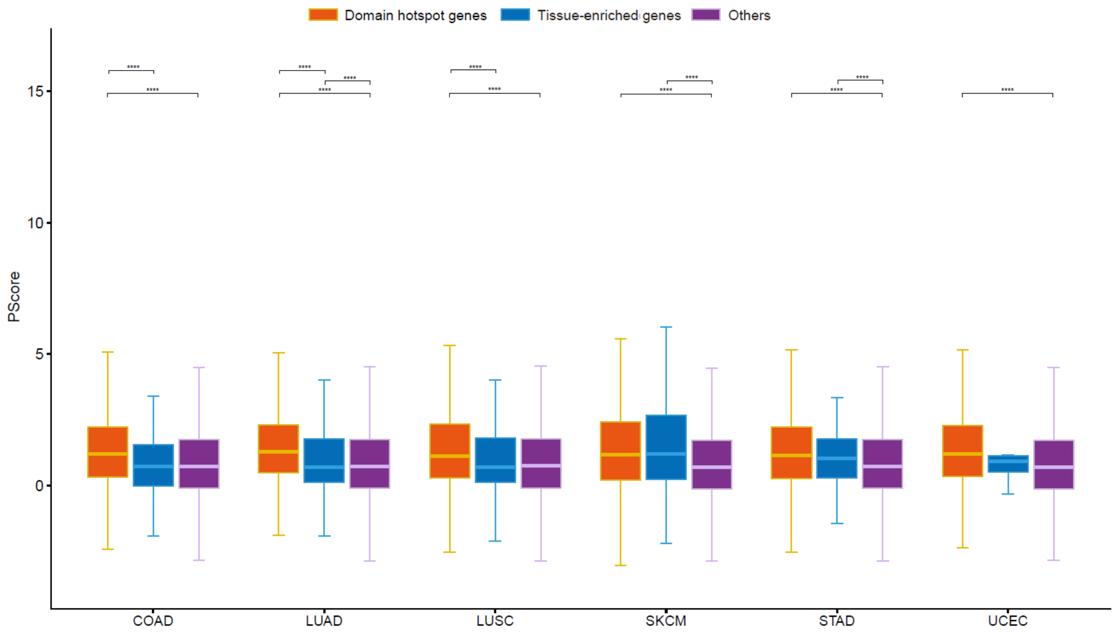

Figure S7. Boxplots showing the PScore of proteins encoded by domain hotspots genes, tissue-enriched genes and other genes across cancer types. Orange, domain hotspots genes; Blue, tissue-enriched genes; Purple, other genes. * p-values < 0.01 for Wilcox's rank sum tests.

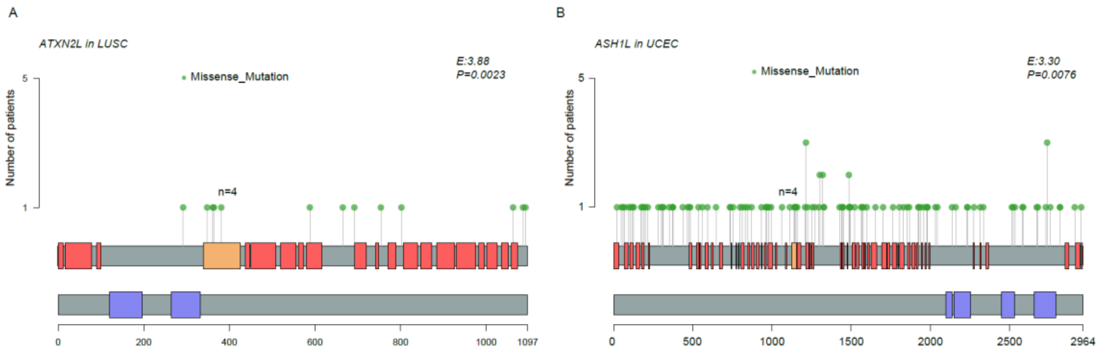

Figure S8. Lollipop plot showing the mutations in ATXN2L and ASH1L genes. A for ATXN2L in LUSC and B for ASH1L in UCEC.

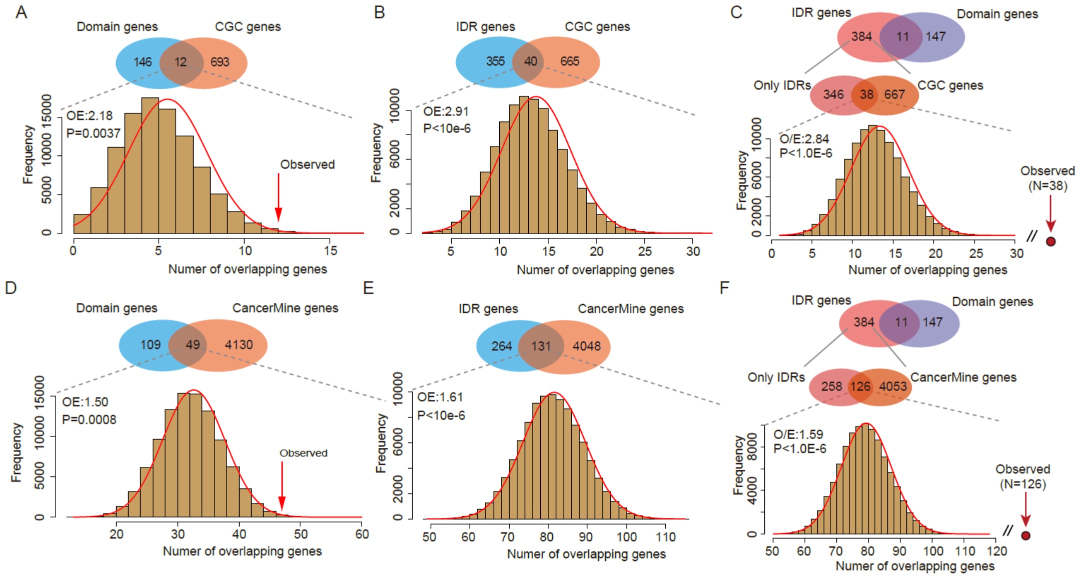

Figure S9. Overlaps of prioritized genes with CGC and CancerMine genes. A for domain genes and CGC genes. B for IDR genes and CGC genes. C for IDR genes and domain genes, the bottom one for only IDR genes and CGC genes. D, for domain genes and CancerMine genes. E for IDR genes and CancerMine genes. F for IDR genes and domain genes, the bottom one for only IDR genes and CancerMine genes.

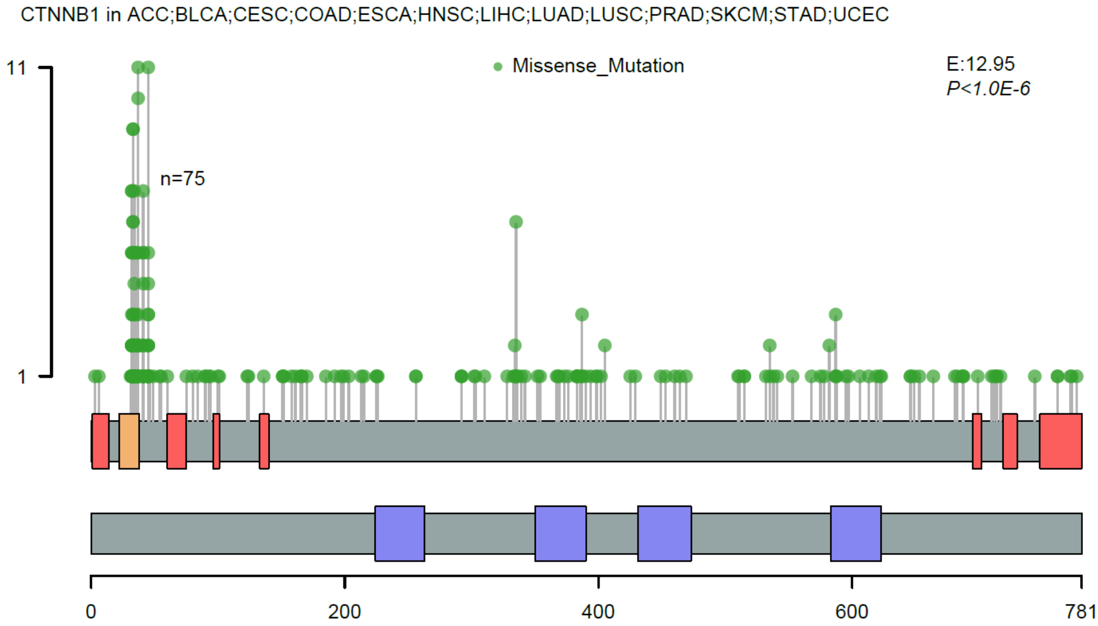

Figure S10. Lollipop plot showing the mutations in CTNNB1 across cancer types.

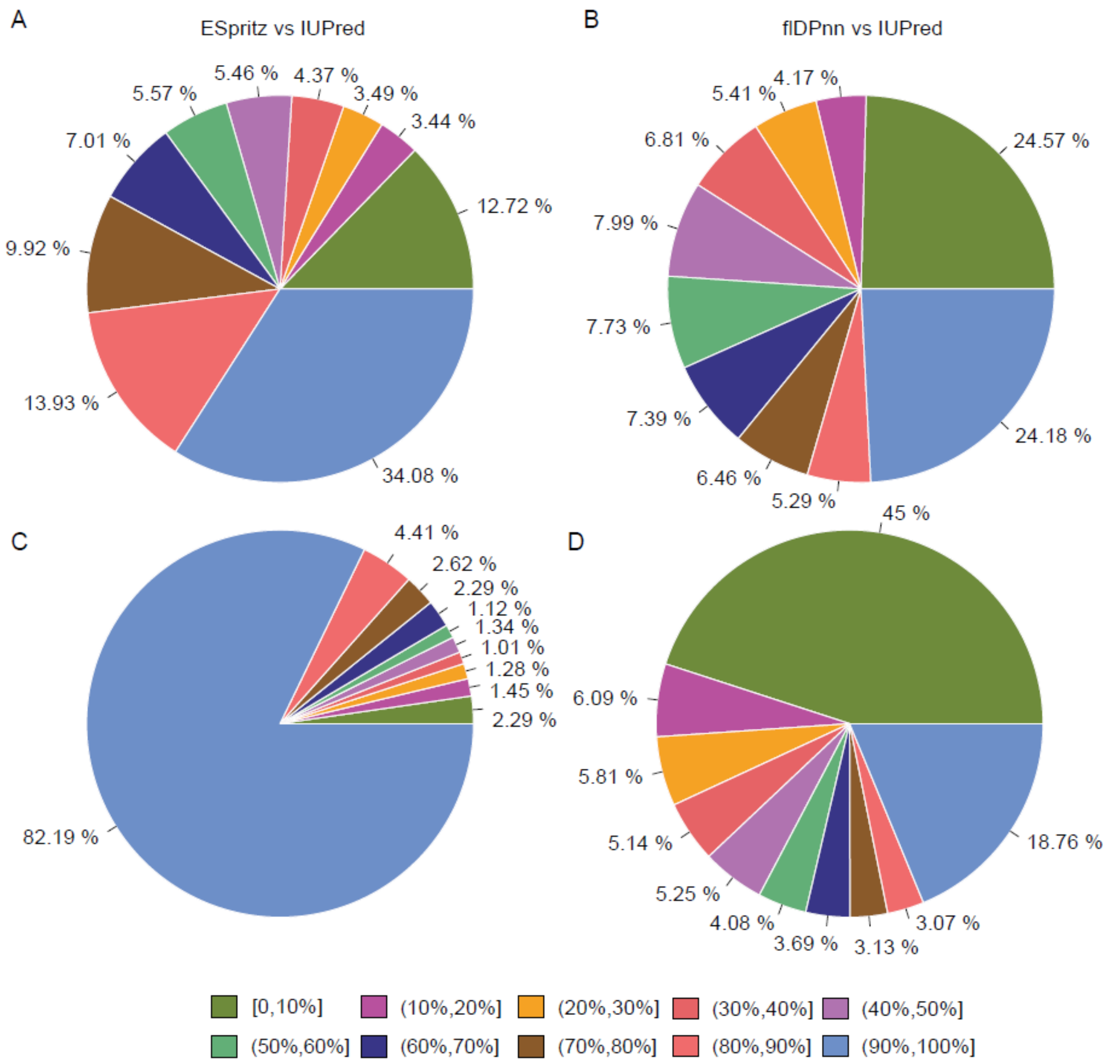

Figure S11. Overlap of IDRs predicted by different methods. A and B, Proportion of all IDRs predicted by IUPred that are supported by ESpritz and fIDPnn. C and D, Proportion of prioritized IDRs predicted by IUPred that are supported by ESpritz and fIDPnn.

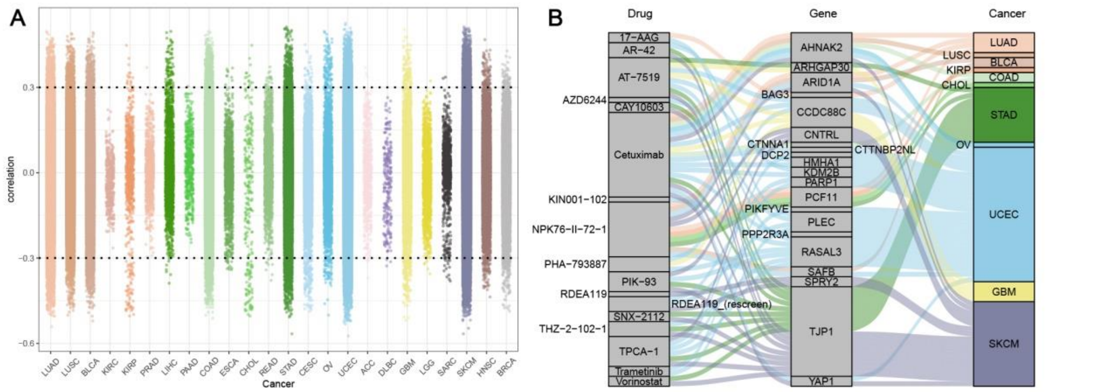

Figure S12. Candidate drugs of which activities are correlated with gene expression. A, Distribution of correlation across cancer types. B, Representative drug-gene-cancer correlations.

Table S1. The number of patients used in this study.

Cancer types	#of patients (mutation)	#of normal samples (expression)	#of cancer samples (expression)
ACC	92	0	79
BLCA	411	19	406
BRCA	1020	113	1014
CESC	289	3	288
CHOL	36	9	36
COAD	406	41	404
DLBC	37	0	37
ESCA	184	11	161
GBM	393	5	159
HNSC	507	44	495
KICH	66	24	65
KIRC	369	72	365
KIRP	281	32	279
LAML	141	0	67
LGG	512	0	521
LIHC	363	50	360
LUAD	567	59	511
LUSC	485	49	482
MESO	82	0	81
OV	412	0	255
PAAD	177	4	170
PCPG	179	3	183
PRAD	497	52	495
READ	150	10	146
SARC	236	2	237
SKCM	466	1	466
STAD	439	32	373
TGCT	145	0	151
THCA	492	58	495
THYM	123	2	118
UCEC	530	35	527
UCS	57	0	56
UVM	80	0	80

Table S2. Prioritized genes enriching mutations within IDRs.
Table S3. Prioritized genes enriching mutations within domains.
